# Protease Inhibitors Extracted from* Caesalpinia echinata* Lam. Affect Kinin Release during Lung Inflammation

**DOI:** 10.1155/2016/9425807

**Published:** 2016-12-01

**Authors:** Ilana Cruz-Silva, Viviane Abreu Nunes, Andrezza Justino Gozzo, Priscila Praxedes-Garcia, Aparecida Sadae Tanaka, Kazuaki Shimamoto, Mariana Silva Araujo

**Affiliations:** ^1^Department of Biochemistry, Universidade Federal de São Paulo, Rua Três de Maio, No. 100, 04044-020 São Paulo, SP, Brazil; ^2^School of Arts, Sciences and Humanities, Universidade de São Paulo, Avenida Arlindo Bettio, No. 1000, 03828-000 São Paulo, SP, Brazil; ^3^Department of Marine Sciences, Universidade Federal de São Paulo, Rua Doutor Carvalho de Mendonça, No. 144, 11070-100 Santos, SP, Brazil; ^4^Japan Health Care College, Sinei 434-1, Kiyota-ku, Sapporo, Japan

## Abstract

Inflammation is an essential process in many pulmonary diseases in which kinins are generated by protease action on kininogen, a phenomenon that is blocked by protease inhibitors. We evaluated kinin release in an* in vivo* lung inflammation model in rats, in the presence or absence of CeKI (*C. echinata* kallikrein inhibitor), a plasma kallikrein, cathepsin G, and proteinase-3 inhibitor, and rCeEI (recombinant* C. echinata* elastase inhibitor), which inhibits these proteases and also neutrophil elastase. Wistar rats were intravenously treated with buffer (negative control) or inhibitors and, subsequently, lipopolysaccharide was injected into their lungs. Blood, bronchoalveolar lavage fluid (BALF), and lung tissue were collected. In plasma, kinin release was higher in the LPS-treated animals in comparison to CeKI or rCeEI groups. rCeEI-treated animals presented less kinin than CeKI-treated group. Our data suggest that kinins play a pivotal role in lung inflammation and may be generated by different enzymes; however, neutrophil elastase seems to be the most important in the lung tissue context. These results open perspectives for a better understanding of biological process where neutrophil enzymes participate and indicate these plant inhibitors and their recombinant correlates for therapeutic trials involving pulmonary diseases.

## 1. Introduction

Lung diseases are responsible for the death of millions of people every year. Inflammation is an essential component of many of these disorders, such as pneumonia, asthma, cancer, chronic obstructive diseases, acute lung injury, and granulomatous lung diseases. In this scenario, kinins are highly important in the physiopathology of lung inflammation. Once kinins are able to induce epithelial cells to release bronchodilators and mucus secretion, they participate in the airway smooth muscle contraction, leading to increased microvascular leakage [1].* In vivo*, the activation of the plasma kallikrein-kinin system occurs when plasma prekallikrein and the HK (high-molecular-weight kininogen) assemble on endothelial cells [2]. Plasma prekallikrein is activated in plasma kallikrein, which releases BK (bradykinin) from HK that in turn stimulates B_2_ receptor. In the tissues, KLK1 (kallikrein 1) hydrolyzes LK (low-molecular-weight kininogen) to release Lys-BK, which is converted into BK by aminopeptidase [3]. Lys-BK also acts on B_2_ receptor. Although this represents the major mechanism to BK release, bioactive kinins are also generated from kininogens by the action of other enzymes such as neutrophil proteases.

During the inflammatory process, neutrophils migrate toward the site of inflammation and degranulate and release proteolytic enzymes, such as NE (neutrophil elastase), Cat G (cathepsin G), and PR3 (proteinase 3). These enzymes are involved in the degradation of extracellular matrix proteins and act on a variety of plasma proteins [4, 5]. In addition, human neutrophils are able to bind components of the kallikrein-kinin system, such as kininogens [6] and plasma prekallikrein [7]. Neutrophils are also able to express KLK1, KLK4, KLK10, KLK13, KLK14, and KLK15 [8] and kinin receptors [9].

Kininogen is also hydrolyzed by NE releasing E-kinin and by PR3 liberating PR3-kinin. E-kinin does not induce smooth muscle contraction but can bind to the B_2_-receptor after processing at the carboxyterminus [10]. PR3-kinin is able to bind and activate B_1_ receptor and exert a hypotensive effect* in vivo* [11]. Despite kinins having an important role in the regulation of pulmonary neutrophil recruitment, they act as pro- or anti-inflammatory agents, depending on the stimulus and animal model [12–16], and kinin release by proteases is not fully understood in pulmonary inflammation.

One way to evaluate the action of proteases in different physiopathological processes* in vivo* is using molecules that can bind to them, such as proteolytic enzyme inhibitors. These inhibitors are distributed among all living organisms, including animals, plants, and microorganisms.

Several protease inhibitors extracted from plants have been studied for their pharmacological potential. Considering the involvement of proteases in lung inflammation and other lung pathologies, exogenous plant protease inhibitors have been tested [17, 18].

In this context, we extracted and purified two different inhibitors from seeds of* C. echinata *(Brazil-wood): (1) CeEI (*C. echinata* elastase inhibitor), a NE, Cat G, PR3, and plasma kallikrein inhibitor [19] and (2) CeKI (*C. echinata* kallikrein inhibitor), a plasma kallikrein [20], Cat G, and PR3 inhibitor. CeKI also inhibits other proteases related to blood coagulation and fibrinolysis and extends the partial activated thromboplastin time without affecting the prothrombin time [20].

In an edema model, using isolated perfused rabbit lungs, CeEI reduced pulmonary arterial pressure and lung weight, and CeKI was less effective [19]. In the present study, we evaluated kinin release using a lung inflammation model in rats in the absence or presence of protease inhibitors from* C. echinata* seeds.

## 2. Material and Methods

Human plasma kallikrein, neutrophil Cat G, NE and PR3, MeO-Suc-A-A-P-V-pNA, H-D-P-F-R-pNA, aprotinin, AEBSF (4-(2-aminoethyl)benzenesulfonyl fluoride hydrochloride), E-64 (trans-epoxysuccinyl-L-leucylamido-(4-guanidino)-butane), and SBTI (soybean trypsin inhibitor) were obtained from Merck KGaA (Darmstadt, Germany). Suc-A-A-P-F-pNA, TPCK (N-p-tosyl-phenylalanine chloromethyl ketone), o-phenanthroline, lisinopril, and LPS (lipopolysaccharide) were purchased from Sigma-Aldrich (St. Louis, MO, USA). BK and Tyr-BK were obtained from Peptide Institute Inc. (Osaka, Japan) and *γ*-globulin was obtained from Intergen (Osaka, Japan). EDTA (ethylenediaminetetraacetic acid) was purchased from Reagen (Rio de Janeiro, RJ, Brazil) and PEG (polyethylene glycol 6000) from Quimesp (Sao Paulo, SP, Brazil). Rabbit anti-BK antibody was prepared according to the method described elsewhere [22]. ACE and Abz-F-R-K(Dnp)-P-OH [23] were kindly supplied by Dr. Adriana K. Carmona from the Department of Biophysics (UNIFESP, Sao Paulo, SP, Brazil). CeKI was purified in our laboratory according to Cruz-Silva et al., 2004; CeEI was purified in our laboratory according to Cruz-Silva et al., 2013, and rCeEI was cloned, expressed, and purified in our laboratory (Cruz-Silva et al., manuscript in preparation). All other chemicals were obtained from commercial sources and were of the best grade available.

This study was approved by the Ethics in Research Committee of the Universidade Federal de Sao Paulo and performed in accordance with the Guide for Care and Use of Laboratory Animals (National Institute of Health Publication number 86-23, Bethesda, MD, USA) and Lineas Directrices Relativas al Alojamento y a los Cuidados de los Animales (European Community Council, 86/609/CEE).

### 2.1. Inhibitory Constants

The inhibitory activities of rCeEI and CeKI were tested on NE (1.0 nM) in 20 mM Tris buffer pH 7.5, those of Cat G (17 nM) in 50 mM Tris buffer pH 8.0, those of PR3 (0.20 *µ*M) in 50 mM Tris buffer, containing 50 mM NaCl pH 7.5, those of plasma kallikrein (4.0 nM) in 20 mM Tris buffer, containing 30 mM NaCl pH 7.5, KLK1 (18 nM), and those of ACE (20 nM) in 20 mM phosphate buffer pH 7.3. The enzymes were preincubated for 10 min at 37°C with increasing amounts of rCeEI or CeKI. The residual activity of the enzymes was determined with the hydrolysis of appropriate substrates, namely, MeO-Suc-A-A-P-V-pNA (0.20 mM), Suc-A-A-P-F-pNA (0.30 mM), MeO-Suc-A-A-P-V-pNA (0.20 mM), H-D-P-F-R-pNA (0.30 mM), H-D-P-F-R-pNA (0.30 mM), and Abz-F-R-K(Dnp)-P-OH (10 *μ*M), respectively. The ensuing *p*-nitroaniline (*p-*NA) release was followed at 405 nm in a SpectraCount plate reader (Packard Instrument Co., Downers Grove, IL, USA). The hydrolysis of the FRET (fluorescence resonance energy transfer) substrate was followed by measuring the fluorescence at *λ*_em_ = 420 nm and *λ*_ex_ = 320 nm in the same plate reader.

The equilibrium dissociation constant (*K*_*i*_) was determined by measuring the residual enzyme activity on substrate hydrolysis. *K*_*i*_ value was calculated by adjusting the experimental points to the equation for a slow-tight binding mechanism [24] using nonlinear fitting by GraFit, Erithacus Software Ltd. (Horley, UK).

### 2.2. Lung Inflammation Model

For the lung inflammation model, we used the methodology described by Duong et al., 2001 [25], with some modifications. Male three-month-old Wistar rats, weighing approximately 250 g, were obtained from the Central Biotery at the Universidade Federal de Sao Paulo. They had free access to food and water and exposure to alternate standardized light/dark periods of 14 and 10 h/day. Animals were randomized into six groups: negative control (*n* = 8), positive control (*n* = 6), 2.6 mg CeKI (*n* = 6), 7.8 mg CeKI (*n* = 5), 0.78 mg rCeEI (*n* = 5), and 2.6 mg rCeEI (*n* = 5). The animals were pretreated by the intravenous injection (tail vein) of 50 mM Tris buffer pH 8.0 (negative or positive controls), CeKI (2.6 or 7.8 mg), or rCeEI (0.84 or 2.6 mg) diluted in this same buffer. These concentrations were chosen based on previous results with CeKI and CeEI in an isolated lung edema rabbit model [19] and considering the rat blood volume. After 20 min, rats were lightly anesthetized with ketamine and xylazine, and afterwards a small incision was made (<0.3 cm); the same buffer (negative control) or 75 *μ*g LPS/animal diluted in the same buffer (positive control, CeKI, and rCeEI groups) was injected via trachea directly into their lungs. Six hours later, rats were once again anesthetized, a thoracotomy was performed, and blood was collected directly via the inferior cava vein by an incision in the presence of 34 mM EDTA. To obtain plasma, blood was incubated on ice for 60 min followed by centrifugation at 270 ×g for 15 min at 4°C. Lungs were taken, cannulated, and washed with 10 mM phosphate buffer pH 7.4 (10 mL). The washing solution was collected and labeled BALF (bronchoalveolar lavage fluid). Washed lungs were then homogenized in 10 mM potassium phosphate buffer (pH 7.4) and centrifuged at 3,000 ×g for 30 min at 4°C.

### 2.3. Protein Evaluation in BALF

Fifty *μ*g of protein mixture present in the BALF was applied in a sodium dodecyl sulfate polyacrylamide electrophoresis (SDS-PAGE) 12% slab gels according to the Laemmli method [26]; the protein bands were stained with Coomassie brilliant blue and compared to the molecular mass markers from Kaleidoscope Prestained Standards (Bio-Rad Laboratories Hercules, CA, USA).

Total protein quantity was calculated according to Bradford [27] using the Bio-Rad Protein Assay (Bio-Rad Laboratories Hercules, CA, USA) following the manufacturer's instructions. The standard curve was performed with bovine albumin solution.

### 2.4. Polymorphonuclear Neutrophils (PMNs) Counts in BALF

Leukocyte recruitment to alveoli was determined in the BALF in the presence of 0.40% Trypan Blue. Total and differential leukocyte cells were counted manually with a Neubauer chamber using an optical microscope.

### 2.5. Extraction of Kinins from BALF, Plasma, and Lung Samples

The kinin extraction from BALF, plasma, or lung was performed according to the method described by Shimamoto et al., 1988 [28], with some modifications [29]. A cocktail of protease inhibitors (24 *µ*M SBTI, 10 *μ*M lisinopril, 10 mM EDTA, 100 *μ*M TPCK, 10 *μ*M E-64, 5.0 mM o-phenanthroline, 1.0 mM AEBSF, and 9.0 *μ*M aprotinin) was added to BALF, plasma, or lungs, and samples were centrifuged at 950 ×g for 15 min at 4°C. Next, ethanol was added to the supernatant (2 : 1, v/v) and mixed for 30 s. After centrifugation at 950 ×g for 15 min at 4°C, the supernatant was transferred to another tube and vacuum-dried at 56°C. Water, acetone, and petroleum ether (1 : 2 : 7, v/v/v) were added to the pellet and mixed for 30 s. The samples were centrifuged at 950 ×g for 15 min at 4°C; the petroleum ether phase with the lipidic content was discarded and the acetone-aqueous phase was vacuum dried at 56°C.

### 2.6. Radioimmunoassay for Kinins

The pellet obtained in Section 2.5 was submitted to kinin radioimmunoassay according to the method described by Shimamoto et al., 1978, with some modifications [21].

The experiment was performed twice and radiation values were converted into kinin released (pg) using a standard curve.

### 2.7. ACE Activity in Lungs

Samples of lungs (5 *µ*L), collected in the absence of lisinopril, were maintained in 50 mM Tris buffer pH 7.4 containing 50 mM NaCl for 5 min at 37°C before the addition of the substrate Abz-F-R-K(Dnp)-P-OH (10 *µ*M) in a final volume of 200 *µ*l. Fluorescence changes were monitored continuously for 30 min at *λ*_ex_ = 320 nm and *λ*_em_ = 420 nm in a SpectraCount plate reader (Packard Instrument Co., Downers Grove, IL, USA). The slope of the generated fluorescence signal was converted into micromoles of substrate hydrolyzed per minute based on a calibration curve obtained from the complete hydrolysis of peptide and adjusted for total protein quantity. This reaction was performed in triplicate and lisinopril (10 *μ*M) was used to confirm ACE activity.

### 2.8. Statistical Analysis

The results are shown as the mean ± SD of two different experiments performed in triplicate. Statistical analyses were performed using one-way ANOVA (analysis of variance) with the commercial program GraphPad Prism Version 5 (GraphPad Software Inc., San Diego, CA, USA).

## 3. Results

### 3.1. Inhibitory Activity of Enzymes Involved in Lung Inflammation

In previous works, we purified and characterized two inhibitors from* Caesalpinia echinata* seeds: CeEI and CeKI. Both inhibitors share similarities, according to Cruz-Silva et al. [19, 20]. Comparing their partial N-terminal sequence (30 amino acids' residues), they are considered members of Kunitz-type family, although they display 9 different amino acid residues between them. Although both appear as a 20 kDa protein by SDS-PAGE, these inhibitors present different retention times from C18 column, which indicates that these inhibitors have variable hydrophobicity degree. Finally, they display distinct inhibitory activity; CeKI is able to inhibit plasma kallikrein [20] in a nanomolar range and Cat G and PR3 are able to inhibit plasma kallikrein in a micromolar range. On the other hand, rCeEI inhibits NE, plasma kallikrein, and Cat G in a nanomolar range and PR3 in a micromolar range. Also, rCeEI blocks Cat G activity 320-fold better than CeKI. Additionally, CeEI was more effective than CeKI in reducing pulmonary edema in isolated rabbit lung. CeEI also prevented hemodynamic (pulmonary artery pressure) alterations in this model but CeKI did not have any effect [19]. Therefore the major difference between these inhibitors is Cat G and proteinase 3 inhibition. It is also important to emphasize that both inhibitors did not block tissue KLK1 and ACE activity. The inhibitory constants are shown in Table 1.

### 3.2. Inflammatory PMN Counts

**Figure 1:**
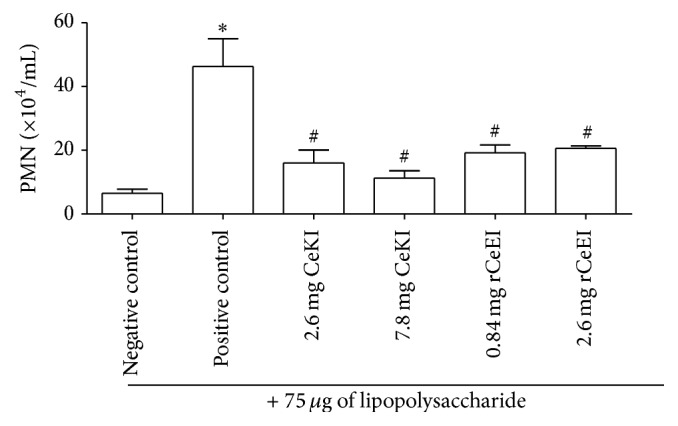
PMN quantification in BALF. Rats were pretreated intravenously with buffer (negative or positive controls), CeKI (7.8 or 2.6 mg), or rCeEI (2.6 or 0.84 mg). After 20 min, they received 75 *μ*g LPS/animal (positive control and CeKI and rCeEI groups) or buffer (negative control) injected via the trachea directly into their lungs. Six hours later, lungs were collected and washed with buffer (BALF). BALF (20 *µ*L) was mixed with 0.4% Trypan Blue. Total and differential leukocyte cells were counted manually with a Neubauer chamber using optical microscopy. ^*∗*^Significant difference compared to negative control (*ρ* < 0.05). ^#^Significant difference compared to positive control (*ρ* < 0.05).

LPS is an endotoxin found in the outer membrane of Gram-negative bacteria and it has a potent proinflammatory activity. In order to verify LPS-induced immune response, we evaluated PMN infiltration into the alveolar space, counting leukocyte cells in BALF. In BALF samples, we verified a 7.1-fold increase in PMNs in the positive control compared to the negative control, which was used as an indication of cell extravasation (Figure 1). All inhibitors were able to diminish the inflammatory cell translocation to the BALF. CeKI (7.8 mg and 2.6 mg groups) reduced by 4.1- and 3.0-fold, respectively, while rCeEI (0.84 mg and 2.6 mg) caused a reduction in PMN amount by 2.5- and 2.1-fold, respectively, indicating that the smaller concentration was already responsible for the maximum effect.

### 3.3. Kinin Release

**Figure 2:**
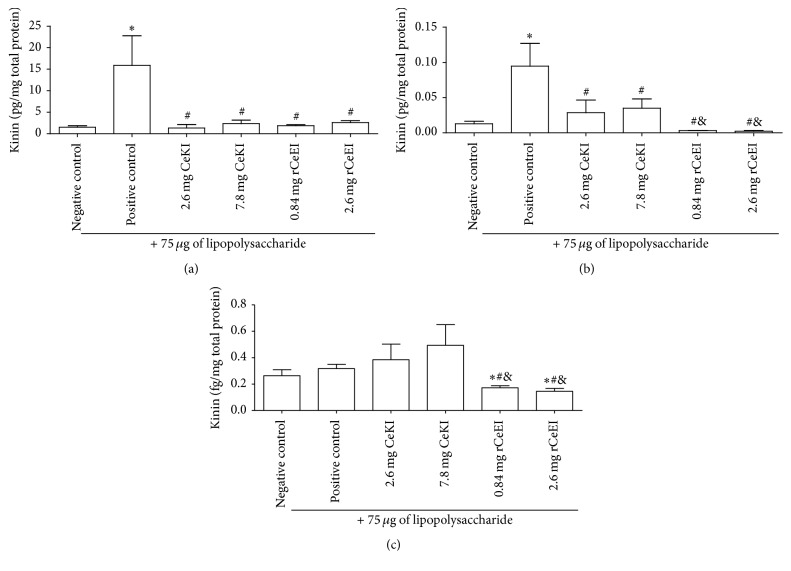
Kinin release in BALF, plasma, and lungs. Rats were pretreated intravenously with buffer (negative or positive controls), CeKI (7.8 or 2.6 mg), or rCeEI (2.6 or 0.84 mg). After 20 min, they received 75 *μ*g of LPS/animal (positive control and CeKI and rCeEI groups) or buffer (negative control) injected via the trachea directly into their lungs. Six hours later, blood was collected, BALF was obtained, and lungs were extracted. Kinin was extracted from BALF (a), plasma (b), or homogenized lung (c) using a treatment with ethanol and then water, acetone, and petroleum ether. For kinin quantification, a radioimmunoassay was performed according to Shimamoto et al., 1978, with some modifications [21]. The experiment was performed twice and radiation values were converted into kinin (pg) using a standard curve. ^*∗*^Significant difference compared to negative control (*ρ*<0.05). ^#^Significant difference compared to positive control (*ρ* < 0.05). ^&^Significant difference compared to CeKI-treated groups (*ρ* < 0.05).

Kinins were extracted by ethanol from BALF, plasma, or lung in the presence of a cocktail of protease inhibitors and submitted to kinin radioimmunoassay. In BALF, kinin release was observed in all groups. The LPS group (positive control) showed high kinin content (15 pg/mg of total protein), which corresponds to an augment of 13.5-fold in comparison to the negative control. The inhibitors reduced kinin release up to 9.0-fold, with no significant difference between experimental groups (Figure 2(a)).

In plasma, kinin release was also detected in all groups. Similarly, in the positive control, there was higher kinin generation (0.1 pg/mg of total protein) than in the negative control (7.5-fold). Accordingly, rCeEI reduced kinin release by 50-fold and CeKI up to 4-fold (Figure 2(b)) in comparison to LPS group. Therefore, kinin in plasma of rCeEI-treated animals was lower than CeKI-treated animals by 13.5-fold in average.

In lungs, we observed smaller kinin release (about fg/mg of total protein) compared to BALF and plasma. In addition, there was a significant reduction in kinin concentration only in rCeEI-treated group compared to the positive control (2.3-fold) (Figure 2(c)), confirming the importance of NE in the process.

**Figure 3:**
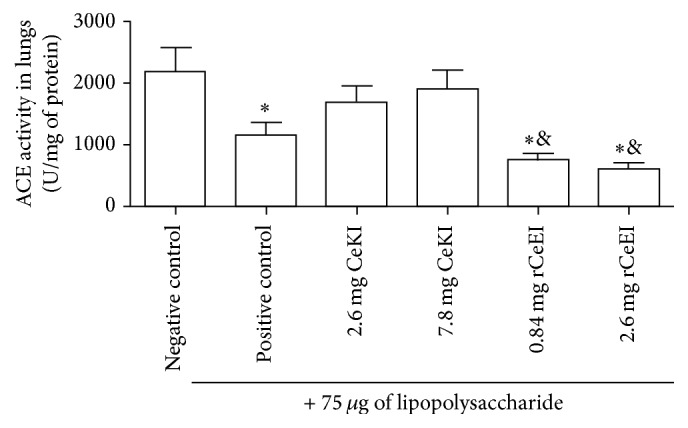
ACE activity in lungs. Rats were pretreated intravenously with buffer (negative or positive controls), CeKI (7.8 or 2.6 mg), or rCeEI (2.6 or 0.84 mg). After 20 min, they received 75 *μ*g of LPS/animal (positive control and CeKI and rCeEI groups) or buffer (negative control) injected via the trachea directly into their lungs. Six hours later, lungs were extracted and homogenized. Samples of lung (5 *µ*L) were maintained in 50 mM Tris buffer at pH 7.4 containing 50 mM NaCl for 5 min at 37°C before the addition of the substrate Abz-F-R-K(Dnp)-P-OH (10 *µ*M) in a final volume of 200 *µ*L. Fluorescence changes were monitored continuously for 30 min at *λ*_ex_ = 320 nm and *λ*_em_ = 420 nm. The slope of the generated fluorescence signal was converted into micromoles of substrate hydrolyzed per minute based on a calibration curve obtained from the complete hydrolysis of peptide and adjusted for total protein quantity. ^*∗*^Significant difference compared to negative control (*ρ* < 0.05). ^&^Significant difference compared to CeKI-treated groups (*ρ* < 0.05).

In order to understand the small presence of kinin in that tissue, we evaluated the kininase activity of ACE. As expected, a high ACE activity in the lung tissue, even in inhibitor-treated animals, was observed (Figure 3). Nevertheless, a significant reduction of ACE activity in rCeEI group was showed. Despite the fact that the ACE activity was shown to be smaller in rCeEI-treated animals, the kinin amount in this group was also low, which indicates that this result was mainly due to lowered kinins by rCeEI compared to reduced ACE.

## 4. Discussion

The underlying pathophysiological mechanisms in lung inflammation require a variety of disrupted homeostatic events involving pulmonary endothelial and epithelial barrier dysfunction, recruitment and neutrophil activation, and the release of potent antimicrobial substances including oxidants and proteases, such as NE, Cat G, and PR3. During inflammation, these enzymes are able to cleave several cellular substrates, and, under pathological conditions, they can damage host tissues.

Here we analyzed the interplay between neutrophil recruitment and protease release, as well as the kallikrein-kinin system activation using LPS-induced lung inflammation model in rats and kinin detection method in BALF, plasma, and lungs.

PMN participation, primarily neutrophils, has been described in many pulmonary diseases. In general, PMN infiltration can be observed from 4 to 8 hours after LPS or Gram-negative bacteria application or inhalation [30, 31]. Our results showed enhanced PMN recruitment into alveolar space (about 7-fold) in LPS group suggesting that this model mimics lung inflammation in which the native immune response is activated. In contrast, inhibitor-treated animals displayed smaller PMN extravasation, with similar behavior to the negative control, indicating that rCeEI and CeKI were efficient in reducing PMN migration into the BALF.

The mechanism used by PMN to cross the endothelium and enter the alveolar space is not completely clear; however leukocyte chemotaxis has been associated with PMN migration [32]. Among chemoattractants, some are related to protease activity. Cat G has been demonstrated as a potent chemoattractant for monocytes and neutrophils* in vitro* [33], as well as in patients with inflammatory disease, such as rheumatoid arthritis [34]. So, it is reasonable to think that neutrophil proteases could mediate chemotactic action for PMN, which is in agreement with the lung inflammation model used here. Additionally, our results with the protease inhibitors CeKI and rCeEI, which individually inhibit kallikrein, PR3, and Cat G, suggested that the inhibition of these enzymes is a key event for reducing PMN migration into the alveolar space. Similarly, Oliveira et al. [35] showed that BbCI, a NE-Cat G inhibitor extracted from* Bauhinia bauhinioides* seeds, strongly inhibited leucocyte rolling, adhesion, and migration, in an inflammation model induced by carrageenan.

Regarding kinin generation, it is well know that this peptide is involved in the major signs of inflammation, such as pain, heat, redness, and swelling [36].

**Figure 4:**
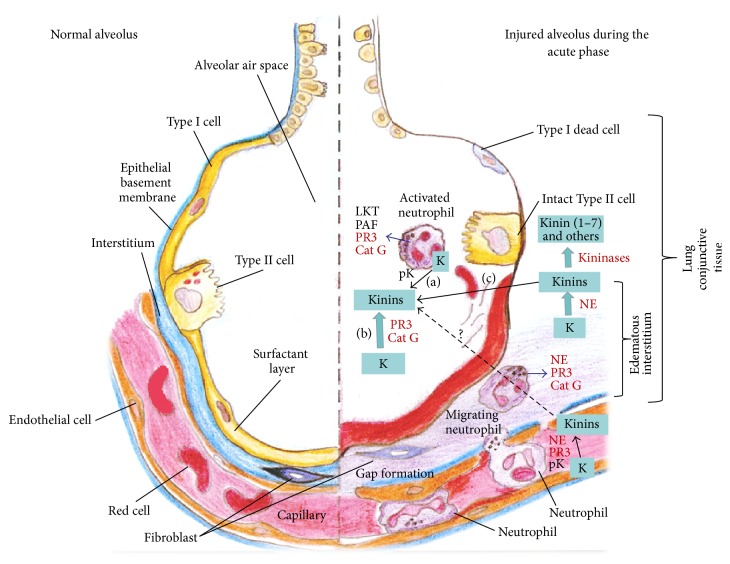
Potential sources of kinin in LPS-induced lung inflammation model. Kinin may be generated in lung via kininogen hydrolysis and is rapidly degraded by kininases. This peptide can be released into the alveolar space through distinct pathways: (a) in neutrophil-bound kininogen cleavage by plasma kallikrein, (b) kininogen hydrolysis by NE and PR3, or (c) diffusion from the lung interstitium to the alveolar space. In the plasma, pK, PR3, and NE all release kinin. Additionally, kinin might be exchanged between plasma, lung, and alveolar space. K: kininogen (high-molecular-weight, low-molecular-weight, and/or T-kininogen); pK: plasma kallikrein; NE: neutrophil elastase; PR3: proteinase 3; BALF: bronchoalveolar lavage fluid; LKT: leukotrienes; PAF: platelet activator factor.

Using this LPS-induced lung inflammation model, we detected different kinin amount in the analyzed compartments (BALF, plasma, and lung), where they are believed to mediate these inflammatory events. Considering all potential sources of kinin and its high quantity in BALF (approximately 15 pg/mg of total protein in LPS group), it can be suggested that this peptide could greatly be produced or be translocated into the alveolar space. In plasma, kinin content was smaller almost 150 times in comparison to BALF, while in lungs kinin was detected in a fg scale (per mg of total protein), which was slighter compared to BALF and plasma.  Figure 4 summarizes the possible kinin-generating pathways in this lung inflammation model.

Typically, kinin generation has been demonstrated in plasma and attributed to HK proteolysis by plasma kallikrein. However, this is not enough to explain the potential sources of kinin in the current model. In this context, Idell et al. [37] showed that kallikrein, prekallikrein, and factor XIa-like activities as well as the HK antigen were found in BALF of patients with ARDS (adult respiratory distress syndrome). In addition, KLK1 and kinin were detected in BALF of asthmatic subjects [38, 39] and antagonists of bradykinin B1 and B2 receptors showed modulatory effects in allergic and immune complex-induced lung inflammation in mice with neutrophil participation [14, 15]. These findings suggest that the kallikrein-kinin cascade may be activated in the lungs of patients during these pathological conditions, although the interplay between kallikrein-kinin system and neutrophil proteases (NE, Cat G, and PR3) in lung inflammation has been poorly investigated.

In this respect, it was demonstrated that kininogen can be also hydrolyzed by PR3 [11] and additionally facilitate kinin system activation as it cleaves and inactivates the C1 inhibitor, the major inhibitor of the kinin system [40]. The proteolytic effect of PR3 on the C1 inhibitor, leading to its inactivation, could result in uncontrolled kinin system activation, which may, in turn, intensify the inflammatory response. Additionally, although little is recognized about Cat G involvement in kinin generation, it is already known that this enzyme is able to form a complex with HK, which interferes in Cat G-induced platelet activation without affecting its amidolytic activity [41].

These evidences indicate that kinin could be generated by different enzymes and scenarios; thus, to better discriminate the action of these enzymes in this lung inflammation model, we used two protease inhibitors extracted from* C. echinata *seeds, CeKI and rCeEI, which differently inhibit plasma kallikrein, PR3, Cat G, and NE. In BALF, both inhibitors reduced kinin release up to 9.0-fold, with no significant difference between experimental groups, indicating that a combination of kinin-generating pathways was activated in that compartment, and the inhibition of neutrophil enzymes (NE, Cat G, and PR3) besides plasma kallikrein was responsible for this result. Although the presence of kallikrein is something unusual in BALF, our results regarding PMN extravasation into alveolar space, in addition to a number of evidences, support this suggestion, as neutrophils are able to bind kininogen [6, 42] and plasma kallikrein [7].

In the plasma, kinin release was mainly reduced by rCeEI up to 50-fold in comparison to LPS group, which may indicate that NE play a central role in kinin generation in that model. In the lungs, the smaller kinin release (about fg/mg of total protein) compared to BALF and plasma was observed. It is absolutely reasonable that the slighter kinin content in lungs is a result of ACE activity, which is abundant on the surface of lung endothelial cells [43] and it was shown to be very high in the experiment for ACE activity detection. It is well known that, during the few seconds of passage in the pulmonary vascular bed, 80% to 95% of the biological activity of BK was eliminated [44]. Accordingly, we propose that part of the generated kinin in the lung was degraded by ACE during their passage through the pulmonary vessels. However, if not metabolized by ACE, kinin produced in the lung might easily enter the alveolus, contributing to the total amount of this peptide in BALF (Figure 4).

In the lung tissue, similarly to other investigated compartments, since there was a significant reduction in kinin concentration in rCeEI-treated group compared to the positive control (2.3-fold), NE seems to be a central enzyme for kinin generation. In fact, although kallikrein is a classical enzyme for kinin release, it has been shown that kininogen can be hydrolyzed by NE and the released E-kinin is subsequently cleaved to produce BK [10, 45]. These evidences support our data, which suggest that kinin may be released from kininogen by neutrophil proteases, especially NE, as the treatment of animals with rCeEI better attenuated this process.

Since protease inhibitors can access the alveolar microenvironment and bind to the major enzymes involved in the inflammation processes, they appear as tools to allow a better understanding of the lung inflammation process and its control. Also, they are good candidates for further investigation using DNA recombinant approaches in order to define the minimal dominium for inhibitory activity.

## Figures and Tables

**Table 1:** Specificities of rCeEI and CeKI on enzymes.

	*Ki* (nM)	
	rCeEI	CeKI
Neutrophil elastase	0.67 ± 0.05	n.i.^*∗*^
Neutrophil cathepsin G	6.54 ± 0.11	2100 ± 630
Neutrophil proteinase 3	3700 ± 67	2700 ± 205
Plasma kallikrein	1.00 ± 0.08	3.1 ± 0.1^*∗*^
Tissue kallikrein 1	n.i.	n.i.
Angiotensin-converting enzyme	n.i.	n.i.

“n.i.”: no detectable inhibition.

^*∗*^Cruz-Silva et al., 2004.

NE (1.0 nM), Cat G (17 nM), PR3 (0.20 *µ*M), plasma kallikrein (4.0 nM), KLK1 (18 nM), and ACE (20 nM) activities were determined by observing the hydrolysis of the appropriate substrates, namely, MeO-Suc-A-A-P-V-pNA (0.20 mM), Suc-A-A-P-F-pNA (0.30 mM), MeO-Suc-A-A-P-V-pNA (0.20 mM), H-D-P-F-R-pNA (0.30 mM), H-D-P-F-R-pNA (0.30 mM), and Abz-F-R-K(Dnp)-P-OH (10 *μ*M), respectively. The enzymes were preincubated in appropriate buffer for 10 min at 37°C with increasing amounts of rCeEI or CeKI. The *p*-nitroaniline release was followed at 405 nm or the fluorescence was measured at *λ*_exc_ = 320 nm and *λ*_em_ = 420 nm and *K*_*i*_ values were calculated.

## Abbreviations

PMNs: Polymorphonuclear neutrophils
NE: Neutrophil elastase
Cat G: Cathepsin G
PR3: Proteinase 3
rCeEI: Recombinant elastase inhibitor from* C. echinata* seeds
CeKI: Plasma kallikrein inhibitor from* C. echinata* seeds
HK: High-molecular-weight kininogen
BK: Bradykinin
KLK1: Tissue kallikrein 1
LK: Low-molecular-weight kininogen
LPS: Lipopolysaccharide
BALF: Bronchoalveolar lavage fluid
ACE: Angiotensin-converting enzyme
*p*-NA: *p*-nitroaniline
MeOSuc: N-Methoxysuccinyl
Abz: Orthoaminobenzoic acid
Dnp: 2,4-Dinitrophenyl
*K*_*i*_: Inhibitory constant.

## References

[1] Barnes P. J. (1992). Bradykinin and asthma. *Thorax*.

[2] Motta G., Rojkjaer R., Hasan A. A. K., Cines D. B., Schmaier A. H. (1998). High molecular weight kininogen regulates prekallikrein assembly and activation on endothelial cells: a novel mechanism for contact activation. *Blood*.

[3] Bhoola K. D., Figueroa C. D., Worthy K. (1992). Bioregulation of kinins: kallikreins, kininogens, and kininases. *Pharmacological Reviews*.

[4] Korkmaz B., Horwitz M. S., Jenne D. E., Gauthier F. (2010). Neutrophil elastase, proteinase 3, and cathepsin G as therapeutic targets in human diseases. *Pharmacological Reviews*.

[5] Korkmaz B., Moreau T., Gauthier F. (2008). Neutrophil elastase, proteinase 3 and cathepsin G: physicochemical properties, activity and physiopathological functions. *Biochimie*.

[6] Figueroa C. D., Henderson L. M., Kaufmann J. (1992). Immunovisualization of high (HK) and low (LK) molecular weight kininogens on isolated human neutrophils. *Blood*.

[7] Henderson L. M., Figueroa C. D., Müller-Esterl W., Bhoola K. D. (1994). Assembly of contact phase factors on the surface of the human neutrophil membrane. *Blood*.

[8] Lizama A. J., Andrade Y., Colivoro P. (2015). Expression and bioregulation of the kallikrein-related peptidases family in the human neutrophil. *Innate Immunity*.

[9] Rajasekariah P., Warlow R. S., Walls R. S. (1997). High affinity bradykinin binding to human inflammatory cells. *Biochemistry and Molecular Biology International*.

[10] Imamura T., Tanase S., Hayashi I., Potempa J., Kozik A., Travis J. (2002). Release of a new vascular permeability enhancing peptide from kininogens by human neutrophil elastase. *Biochemical and Biophysical Research Communications*.

[11] Kahn R., Hellmark T., Leeb-Lundberg L. M. F. (2009). Neutrophil-derived proteinase 3 induces kallikrein-independent release of a novel vasoactive kinin. *The Journal of Immunology*.

[12] Bandeira-Melo C., Calheiros A. S., Silva P. M. R., Cordeiro R. S. B., Teixeira M. M., Martins M. A. (1999). Suppressive effect of distinct bradykinin B2 receptor antagonist on allergen-evoked exudation and leukocyte infiltration in sensitized rats. *British Journal of Pharmacology*.

[13] Eric J., Gabra B. H., Sirois P. (2003). Implication of the bradykinin receptors in antigen-induced pulmonary inflammation in mice. *British Journal of Pharmacology*.

[14] Gama Landgraf R., Steil A. A., Sirois P., Jancar S. (2004). Modulation of allergic and immune complex-induced lung inflammation by bradykinin receptor antagonists. *Inflammation Research*.

[15] Landgraf R. G., Sirois P., Jancar S. (2003). Differential modulation of murine lung inflammation by bradykinin B1 and B2 selective receptor antagonists. *European Journal of Pharmacology*.

[16] Perron M.-S., Gobeil F., Pelletier S., Regoli D., Sirois P. (1999). Involvement of bradykinin B_1_ and B_2_ receptors in pulmonary leukocyte accumulation induced by Sephadex beads in guinea pigs. *European Journal of Pharmacology*.

[17] Neuhof C., Oliva M. L., Maybauer D. (2003). Effect of plant Kunitz inhibitors from *Bauhinia bauhinioides* and *Bauhinia rufa* on pulmonary edema caused by activated neutrophils. *Biological Chemistry*.

[18] Ribeiro J. K. C., Cunha D. D. S., Fook J. M. S. L. L., Sales M. P. (2010). New properties of the soybean trypsin inhibitor: inhibition of human neutrophil elastase and its effect on acute pulmonary injury. *European Journal of Pharmacology*.

[19] Cruz-Silva I., Neuhof C., Gozzo A. J. (2013). Using a *Caesalpinia echinata* Lam. protease inhibitor as a tool for studying the roles of neutrophil elastase, cathepsin G and proteinase 3 in pulmonary edema. *Phytochemistry*.

[20] Cruz-Silva I., Gozzo A. J., Nunes V. A. (2004). A proteinase inhibitor from *Caesalpinia echinata* (pau-brasil) seeds for plasma kallikrein, plasmin and factor XIIa. *Biological Chemistry*.

[21] Gozzo A. J., Nunes V. A., Carmona A. K. (2002). Glycosaminoglycans affect the action of human plasma kallikrein on kininogen hydrolysis and inflammation. *International Immunopharmacology*.

[22] Shimamoto K., Ando T., Nakao T., Tanaka S., Sakuma M., Miyahara M. (1978). A sensitive radioimmunoassay method for urinary kinins in man. *Journal of Laboratory and Clinical Medicine*.

[23] Araujo M. C., Melo R. L., Cesari M. H., Juliano M. A., Juliano L., Carmona A. K. (2000). Peptidase specificity characterization of C- and N-terminal catalytic sites of angiotensin I-converting enzyme. *Biochemistry*.

[24] Morrison J. F. (1982). The slow-binding and slow, tight-binding inhibition of enzyme-catalysed reactions. *Trends in Biochemical Sciences*.

[25] Duong M., Simard M., Bergeron Y., Bergeron M. G. (2001). Kinetic study of the inflammatory response in *Streptococcus pneumoniae* experimental pneumonia treated with the ketolide HMR 3004. *Antimicrobial Agents and Chemotherapy*.

[26] Laemmli U. K. (1970). Cleavage of structural proteins during the assembly of the head of bacteriophage T4. *Nature*.

[27] Bradford M. M. (1976). A rapid and sensitive method for the quantitation of microgram quantities of protein utilizing the principle of protein-dye binding. *Analytical Biochemistry*.

[28] Shimamoto K., Ando T., Tanaka S., Imura O. (1988). The determination of kinin and glandular kallikrein in human biological fluids. *Atemwegs- und Lungenkrankheiten. Jahrgang*.

[29] Malavazi-Piza K. C., Araújo M. S., Godinho R. O., Tanaka A. S. (2004). Effect of invertebrate serine proteinase inhibitors on carrageenan-induced pleural exudation and bradykinin release. *International Immunopharmacology*.

[30] Asti C., Ruggieri V., Porzio S., Chiusaroli R., Melillo G., Caselli G. F. (2000). Lipopolysaccharide-induced lung injury in mice. I. Concomitant evaluation of inflammatory cells and haemorrhagic lung damage. *Pulmonary Pharmacology and Therapeutics*.

[31] Shimada M., Tsukada H., Ishizuka O. (2000). Lipopolysaccharide tolerance in relation to intrabronchial influx of neutrophils in the rat. *Lung*.

[32] Wu Y., Stabach P., Michaud M., Madri J. A. (2005). Neutrophils lacking platelet-endothelial cell adhesion molecule-1 exhibit loss of directionality and motility in CXCR2-mediated chemotaxis. *The Journal of Immunology*.

[33] Chertov O., Ueda H., Xu L. L. (1997). Identification of human neutrophil-derived cathepsin G and azurocidin/CAP37 as chemoattractants for mononuclear cells and neutrophils. *The Journal of Experimental Medicine*.

[34] Miyata J., Tani K., Sato K. (2007). Cathepsin G: the significance in rheumatoid arthritis as a monocyte chemoattractant. *Rheumatology International*.

[35] Oliveira C., Navarro-Xavier R. A., Anjos-Vallota E. A. (2010). Effect of plant neutrophil elastase inhibitor on leucocyte migration, adhesion and cytokine release in inflammatory conditions. *British Journal of Pharmacology*.

[36] Elliott D. F., Horton E. W., Lewis G. P. (1960). Actions of pure bradykinin. *The Journal of Physiology*.

[37] Idell S., Kucich U., Fein A. (1985). Neutrophil elastase-releasing factors in bronchoalveolarlavage from patients with adult respiratory distress syndrome. *The American Review of Respiratory Disease*.

[38] Christiansen S. C., Proud D., Cochrane C. G. (1987). Detection of tissue kallikrein in the bronchoalveolar lavage fluid of asthmatic subjects. *The Journal of Clinical Investigation*.

[39] Christiansen S. C., Proud D., Sarnoff R. B., Juergens U., Cochrane C. G., Zuraw B. L. (1992). Elevation of tissue kallikrein and kinin in the airways of asthmatic subjects after endobronchial allergen challenge. *American Review of Respiratory Disease*.

[40] Leid R. W., Ballieux B. E. P. B., van der Heijden I. (1993). Cleavage and inactivation of human C1 inhibitor by the human leukocyte proteinase, proteinase 3. *European Journal of Immunology*.

[41] Selim T. E., Ghoneim H. R., Abdel Ghaffar H. A., Colman R. W., Dela Cadena R. A. (2001). High molecular mass kininogen inhibits cathepsin G-induced platelet activation by forming a complex with cathepsin G. *Hematology Journal*.

[42] Gustafson E. J., Schmaier A. H., Wachtfogel Y. T., Kaufman N., Kucich U., Colman R. W. (1989). Human neutrophils contain and bind high molecular weight kininogen. *The Journal of Clinical Investigation*.

[43] Ferreira S. H., Vane J. R. (1967). The disappearance of bradykinin and eledoisin in the circulation and vascular beds of the cat. *British Journal of Pharmacology*.

[44] Regoli D., Barabé J. (1980). Pharmacology of bradykinin and related kinins. *Pharmacological Reviews*.

[45] Kozik A., Moore R. B., Potempa J., Imamura T., Rapala-Kozik M., Travis J. (1998). A novel mechanism for bradykinin production at inflammatory sites: diverse effects of a mixture of neutrophil elastase and mast cell tryptase versus tissue and plasma kallikreins on native and oxidized kininogens. *The Journal of Biological Chemistry*.

